# 431. Engraftment of donor derived anaerobic taxa and durable long-term multidrug resistant organism decolonization following fecal microbiota transplantation

**DOI:** 10.1093/ofid/ofae631.145

**Published:** 2025-01-29

**Authors:** Ahmed Babiker, Catherine Brink, Sarah Lohsen, Julia Van Riel, Amanda F Strudwick, Colleen S Kraft, Sarah W Satola, Kostas Konstantinidis, Michael H Woodworth

**Affiliations:** Emory University, Atlanta, GA; Georgia Institute of Technology, Atlanta, Georgia; Emory University School of Medicine, Atlanta, Georgia; Emory University School of Medicine, Atlanta, Georgia; Emory University School of Medicine, Atlanta, Georgia; Emory University School of Medicine, Atlanta, Georgia; Emory University School of Medicine, Division of Infectious Diseases, Atlanta, Georgia; Georgia Institute of Technology, Atlanta, Georgia; Emory University, Atlanta, GA

## Abstract

**Background:**

Microbiome restoration through fecal microbiota transplantation (FMT) is a promising decolonization approach that leads to reduction in multidrug resistant organism (MDRO) colonization and infections. The precise mechanisms by which FMT reduces MDRO colonization are not well elucidated, and long-term follow up (LTF) data from patients treated with FMT for MDRO decolonization are lacking. We enrolled participants of a recently conducted trial of FMT for MDRO decolonization (**PREMIX**, NCT02922816) to a LTF study to estimate long-term engraftment dynamics of donor derived anaerobic taxa and their association with MDRO decolonization.

**Methods:**

Participants provided stool every 6 months for up to 5 years. Stool underwent selective culturing for MDROs by plating on selective chromogenic agars, and confirmation by MALDI-TOF and vitek2. MDROs underwent whole genome sequencing (WGS), and stool samples underwent metagenomic sequencing (mNGS). WGS resolved mNGS tracking of MDROs and metagenome-assembled genomes (MAG) of donor anaerobic taxa was performed. Pairwise comparison of baseline and long-term follow up (LTF) MDRO isolates was performed with a unique strain defined as 99.95% average nucleotide identify difference.

**Results:**

Six PREMIX participants were enrolled to the LTF study, five of whom received FMT and one control. 80% (4/5) of FMT treated patients have remained decolonized (culture negative) while 0% (1/1) of control patients have remained decolonized at most recent follow up (median[IQR] 3.6 [3.4,4.0] years). For the FMT treated patient who had an MDRO detected on LTF sample, the MDRO represented a new acquisition (after broad-spectrum antibiotic exposure close to time of FMT), while the MDRO detected in LFT sample of the control patient was the same MDRO detected at baseline. mNGS revealed persistence of donor anaerobic strains in LTF samples (*Agathobaclulum ammoniilyticum, Akermanisa muciniphila*, *Alistipes putredinis*, *Bacteroides uniformis, Barnesiella intestinihominis*, *Eubacterium xylanophilum*, *Gemmer formicilis*), indicative of engraftment and persistence.

**Conclusion:**

FMT was associated with long-term durable decolonization potentially mediated by the engraftment and persistence of donor derived anaerobic strains.

**Disclosures:**

**Ahmed Babiker, MBBS**, Beckman Coulter Inc.: Advisor/Consultant **Colleen S. Kraft, MD, MSc**, Ferring/Rebiotix: Advisor/Consultant|Seres LLC: Advisor/Consultant

